# Shielding the Achilles Heel of Atrial Fibrillation Ablation

**DOI:** 10.1016/s0972-6292(16)30541-1

**Published:** 2012-09-01

**Authors:** B Hygriv Rao

**Affiliations:** Senior Consultant Cardiologist and Electrophysiologist, KIMS Hospitals, Hyderabad

**Keywords:** atrial fibrillation, ablation

Collateral damage to esophagus with ablative therapies for atrial fibrillation (AF) remains a major concern with percutaneous catheter based therapies. Endoscopically documented thermal injuries to the esophageal mucosa such as ulcerations or hemorrhages are seen in a significant percentage of patients undergoing AF ablation. [1,2] The reporting of occurrence of a fatal left atrio-oesophageal fistula (LAEF) with circumferential pulmonary vein isolation in 2004, led to an explosion of innovations and strategies to protect the esophagus during transmural posterior left atrial lesions. The use of low energy lesions in the posterior wall, introduction of a temperature probe in the esophagus, use of proton pump inhibitors, pre and intra procedure imaging and alternative sources of ablation have all contributed to this endeavor. Clinical applications of each of these strategies have shown that none of them are infallible in completely attenuating the vulnerability of esophagus during pulmonary vein isolation.

The introduction of balloon based high intensity focused ultrasound therapy (HIFU) was received with considerable enthusiasm, primarily due its ability to deliver therapy without tissue contact, and absence of requirement for 3 dimensional mapping systems. [3] With the availability of a steerable balloon, increased accessibility of pulmonary veins was achieved resulting in increased procedural success rates compared to earlier generation balloons. The occurrence of LAEF and persistent phrenic nerve palsy with HIFU balloon was a significant setback to this technology, translating into serious impediment to its routine clinical usage.[4] Adherence to a safety algorithm which included culmination of lesions when temperature probe in the esophagus showed a temperature ≥ 40ºC, also could not prevent the lethal esophageal injuries.[5]

The burden of evidence points to the mobile anatomy of the retro cardiac esophagus being responsible for the high prevalence of thermal injuries leading to LAEF which is fortunately a rare but nevertheless catastrophic complication of AF ablation. The proximity of esophagus to posterior left atrium which is accentuated in supine position contributes to mucosal thermal injury, particularly because of absence of serosa in this portion of the gut. The mobile and variable location of the esophagus with respect to each of the pulmonary veins and the source of energy is critical in determining its predilection to injury. The study by Naven et al in this issue of the journal elegantly shows the inverse relation of the luminal temperature of the esophagus to the distance from HIFU balloon. [6] The remoteness of various pulmonary veins in the orthogonally opposite fluoroscopic views to the esophagus dictates the impact of injury during their respective isolation. Though there is no equivocal agreement whether endoluminal temperature recording in the esophagus accurately predicts occurrence of mucosal injury, yet at this point this is the only available monitoring. Optimal use of this tool with the understanding of the dynamically changing distances between the source and target during ablation may contribute to enhancing safety of the procedure. Most of the strategies aimed at protecting the esophagus are mostly energy reducing measures which may compromise the efficacy of therapy. The deficiency of reliable esophageal protection measures are bound to have a frictional effect on growth of AF ablation therapies. Utilization of data from imaging techniques and integrating into mathematical equations to arrive at "safe" distances from ablation sources may comprise future strategies to prevent esophageal injuries in AF ablation.
